# Medical compression therapy of the extremities with medical compression stockings (MCS), phlebological compression bandages (PCB), and medical adaptive compression systems (MAC): S2k guideline of the German Phlebology Society (DGP) in cooperation with the following professional associations: DDG, DGA, DGG, GDL, DGL, BVP

**DOI:** 10.1007/s00105-020-04706-z

**Published:** 2021-01-01

**Authors:** E. Rabe, E. Földi, H. Gerlach, M. Jünger, G. Lulay, A. Miller, K. Protz, S. Reich-Schupke, T. Schwarz, M. Stücker, E. Valesky, F. Pannier

**Affiliations:** 1 Emeritus Klinik und Poliklinik für Dermatologie und Allergologie, Universitätsklinikum Bonn (AöR), Venusberg-Campus 1, 53127 Bonn, Germany; 2 Földiklinik, Rösslehofweg 2–6, 79856 Hinterzarten, Germany; 3 Zehntstr. 25, 68519 Viernheim, Germany; 4 Klinik und Poliklinik f. Hautkrankheiten, Universitätsmedizin, Ferdinand Sauerbruchstraße, 17475 Greifswald, Germany; 5 Klinik für Gefäß- u. Endovaskularchirurgie, Phlebologie-Lymphologie, Frankenburgstr. 31, 48431 Rheine, Germany; 6 Dermatologische Praxis, Wilmersdorfer Str. 62, 10627 Berlin, Germany; 7 Wundforschung, Universitätsklinikum Hamburg-Eppendorf, Bachstr. 75, 22083 Hamburg, Germany; 8 Privatpraxis für Haut- und Gefäßmedizin, Wundtherapie, Hertener Str. 27, 45657 Recklinghausen, Germany; 9 Praxis für Gefäßmedizin, Konrad Goldmann Str. 5b, 79100 Freiburg, Germany; 10 Klinik für Dermatologie, Venerologie und Allergologie, St. Josef Hospital, Ruhr-Universität Bochum, Gudrunstraße 56, 44791 Bochum, Germany; 11 Klinik für Dermatologie, Venerologie und Allergologie, Universitätsklinikum Frankfurt, Theodor-Stern-Kai 7, 60590 Frankfurt, Germany; 12 Praxis für Dermatologie & Phlebologie, Helmholtzstr. 4–6, 53123 Bonn, Germany; 13 Dermatologische Universitätsklinik Köln, Cologne, Germany

The present guidelines comprise relevant aspects of the use of compression therapy with medical compression stockings (MCS), phlebological compression bandages (PCB), and medical adaptive compression systems (MAC) based on an extensive literature search based on the state of scientific knowledge as of December 2018.

These guidelines were prepared by experts within the framework of an electronic consensus process and a consensus conference which took place in Bielefeld, Germany, on September 27, 2018, on the initiative of the German Society of Phlebology (DGP) and the Professional Association of Phlebologists (BVP). The guidelines were adopted by the boards and advisory councils of the DGP and the BVP, and of the participating professional associations, after preparation by the group of experts and extensive debate, on December 31, 2018.

These guidelines do not cover compression therapy with medical thrombosis prophylaxis stockings (MTPS) or with intermittent pneumatic compression (IPC), which are treated in other guidelines (AWMF 003-001, S3; AWMF 037-001, S1).

The recommendations of the AWMF guidelines “Diagnostics and Treatment of Lymphedema” (registration number 058-001) and “Lipedema” (registration number 037-012) shall also be taken into account where appropriate: https://www.awmf.org/uploads/tx_szleitlinien/058-001l_S2k_Diagnostik_und_Therapie_der_Lymphoedeme_2017-05.pdf, https://www.awmf.org/uploads/tx_szleitlinien/037-012l_S1_Lipoedem_2016-01.pdf.

## 1 Definitions

Therapy with medical compression stockings (MCS) or phlebological compression bandages (PCB) is indispensable in the treatment of phlebological and lymphological diseases of the arms and legs. MCS are stocking-shaped knitted elastic garments; the effect of PCB is achieved by the application of elastic and/or inelastic bandages. Prepared bandaging systems (known as multiple-component systems), medical adaptive compression systems (MAC), and multi-layer stocking systems (known as ulcer stocking systems) are also available for leg ulcer therapy [1].

MCS and PCB have elastic properties, exercising continuous defined pressure on the limb. They increase venous and lymphatic drainage and improve the muscle pump function. While PCB (with bandages and bandage systems) and MAC are usually applied in the decongestion phase, MCS and ulcer stocking systems are used in the long-term therapy and maintenance phases, and MCS in prevention.

PCB can be designed as changeable or permanent bandages. A changeable bandage is replaced daily, and in the best case also left on overnight. Permanent bandages on the other hand, e.g., with multiple-component systems, are left on for longer, generally several days, and not removed at night. PCB generally include the foot and ankle and go up as high as either the head of the fibula or the proximal thigh.

Individual bandages shall be distinguished from bandage systems. The elastic properties of the final bandage system, and thus its stiffness, can be altered by wrapping bandages over one another in several layers and by the use of different materials. In this way, a PCB consisting of several layers of individual elastic bandages can finally assume the properties of inelastic bandaging on the leg. The same is true of adhesive and cohesive bandages. In any case, a PCB consisting of several components or layers is better for healing than one consisting of just one component or layer [1–3]. Medical adaptive compression systems (MAC) are also called adjustable compression wrap devices. They consist of mostly inelastic materials and are fixed with Velcro or equivalents such as hooks. When applied, these systems present high stiffness. They can include separate components for the foot, calf, and thigh, and for the hand and arm. If appropriate, MAC can be fitted independently by the patient if he/she is still sufficiently mobile; however, the hook-tape system also makes fitting the bandages easier for therapists, relatives, or carers. This encourages independence in the patient and supports adherence.

The use of medical compression therapy requires special knowledge and experience, not only for diagnosis, differential diagnosis, risks, and contraindications, but also for ordering compression materials opportunely and for the application technique.

The patient’s age and the condition of his/her skin, musculature, and connective tissue all play a part in the selection of the compression material. The tissue of geriatric patients is often very sensitive, so the material and the pressure shall be adapted to their conditions.

### Recommendation 1

Medical compression therapy shall be an integral component of the treatment of phlebological disease. It can be applied by MCS, PCB, or MAC. Special knowledge and experience are necessary, not only for diagnosis, differential diagnosis, risks, and contraindications, but also for ordering contemporary compression materials and for the application technique.

### 1.1 Pressure and stiffness

The effectiveness of compression therapy is dependent on the skill of the person applying the system, the pressure exerted by the compression device, the number of components and layers used, and the materials of which they are made. By “pressure at rest,” we understand the pressure exerted by the compression device when the patient is lying quietly. When the patient stands up, or the muscles are activated by walking, the pressure increases. This is called “work pressure.” As a rule, the pressure prescribed is the pressure at rest. Thus, the compression classes of MCS refer to the pressure in the ankle area under rest conditions.

According to Laplace’s law, the pressure is proportional to the tension of the compression material, but also to the radius of the tissue under the bandage (leg, arm). The limbs present inhomogeneous radii, with small (ankle bones, Achilles tendon, tibia edge, fibula head, edge of the foot) and large radii (thigh, Bisgaard’s coulisse). To maintain adequate pressure, small radii (e.g., right and left of the Achilles tendon) and large radii (e.g., leg ulcer in Bisgaard’s coulisse) should be padded individually as appropriate with special padding material. This will prevent pressure sores, ensure regular pressure distribution, and improve healing.

#### Recommendation 2

Because the pressure is inversely proportional to the radius, differences in circumference under the compression system shall be padded appropriately in critical cases (e.g., cachexia), with small radii (e.g., ankle region), and with unusual alterations in the circumference of the leg (e.g., Bisgaard’s coulisse, Achilles tendon). This will increase the effectiveness of the treatment on the one hand and avoid pressure sores on the other.

It is also decisive for the clinical and hemodynamic effectiveness of the compression system to determine at what increase in circumference the compression device resists if the patient is moving, if an edema forms, or when the patient stands up, leading to increased pressure under the compression system. This property of the compression material is called stiffness. If the material stretches easily, the pressure increase is small, whereas it can be very large if the material stretches very little or not at all. The pressure increase under the compression device when the patient stands up from the lying position is called the static stiffness index (SSI) [4]. The SSI of different materials at the same initial pressure can vary widely.

#### Recommendation 3

When selecting and ordering compression materials, both the required pressure and the most suitable material should be considered. The effect of the compression system depends not only on the pressure but also on the properties of the material.

## 2 Medical compression devices

### 2.1 Medical compression stockings (MCS)

#### 2.1.1 Types of knit

Medical compression stocking are made in different knits:

- *Flat-knit with seams*, machine-made, with at least one knitted thread and one inserted elastic thread in every second course. These stockings can be produced to fit very closely and apply a high level of compression. Commercially available flat knit MCS are generally stiffer; however, they are also more inflexible, making them harder to slip on over folds of tissue. These properties (greater stiffness + inflexibility + harder to slip on) are not found in circular-knit stockings. In flat-knit stockings, individual courses can be added or eliminated. They can therefore be adapted to unusual leg circumferences, e.g., with highly developed lymphedema. The stocking is knitted as a single piece and stitched together, with a seam at the back of the leg.
- *Single- and double-surface circular-knit with seams*, machine-made, with at least one knitted thread and one inserted elastic thread in every second course. These MCS are produced with a specific number of needles on a knitting cylinder. Additional courses cannot be either added or eliminated. The stocking can only be adapted to the shape of the leg by altering the size of the course (tight or loose knitting) or the tension of the threads. There are therefore limitations on the shaping of circular-knit MCS. Limbs with very small circumferences and extreme changes in circumference or deep folds of tissue cannot be provided with circular-knit stockings.

##### Recommendation 4

Generally, flat-knit quality shall be ordered for limbs with relatively large changes in the circumference, conical shape, or deep folds of tissue, since circular-knit material is not suitable for certain anatomical proportions. For example, very marked alterations in circumference or deep folds of tissue may be found along the arms and legs of patients with chronic venous incompetence and pronounced lymph- or lipedema, as well as adipose patients.

##### Recommendation 5

Because of the type of knit, flat-knit MCS generally have greater stiffness but also greater inflexibility. These properties should be used to treat patients with lymphedema or lipedema, severe chronic venous incompetence (CVI), or adiposity, and also neuropathies and arterial occlusive disease, to avoid pressure peaks due to constriction.

#### 2.1.2 Quality features

MCS should comply with the quality features set out in the relevant guidelines.

##### Recommendation 6

MCS shall comply with the valid RAL GZG 387 (2000) and guideline 93/42 EWG of the European Council and qualify as a dual-tension stocking, i.e., sufficiently elastic lengthwise and crosswise.

##### 2.1.2.1 Stocking models and lengths.

MCS for legs produced according to the standard, called ready-made, series, or standard stockings, are offered in four models:

- Calf stockings (A–D)
- Knee-length stockings (A–F)
- Thigh stockings (A–G)
- Compression support tights (A–T)

They all come in defined lengths (A to D–T). The standard prescribes three lengths for each model, to fit short, normal, and long legs.

The choice of length depends on the diagnosis and the location of the symptoms and alterations. Calf stockings are often sufficient for lower leg edemas, skin alterations in a context of CVI, and venous leg ulcer or ulcus cruris venosum (UCV). Thigh-length stockings or support tights are generally required with lymphedemas and edemas above the knee or of the whole leg after iliac vein thrombosis. The panty part of the support tights can be made with very little pressure to simply support the garment, or with higher pressures for treating edema (e.g., pelvic lymphedema).

Arm stockings are also available to treat symptoms in the upper limbs:

- with or without a shoulder cap
- with or without an incorporated or separate glove

Special made-to-measure compression devices are available to treat toe and finger edemas.

##### Recommendation 7

When ordering MCS, it shall be remembered that different lengths and special parts are available. The choice of length and auxiliary elements depends on the diagnosis and the location of the symptoms and alterations.

Auxiliary elements are available for flat-knit MCS to treat special problem locations, both in terms of garment support and the compression element (e.g., oblique closure, pockets for padding/pelottes, wide opening).

##### Recommendation 8

In treatments with flat-knitted MCS, use should be made of the possibilities offered by the auxiliary elements to obtain a better therapeutic outcome.

##### 2.1.2.1.1 Ulcer compression stockings.

Two-layer ulcer compression stocking systems are available for treating leg ulcers with MCS. They usually consist of a thinner understocking, which can be kept on at night, and a stronger stocking with compression which is worn on top. On the one hand, the combination of the two types of stocking makes it easier to use dressings, and on the other, the use of two layers increases the stiffness of the compression system. The pressure at rest is generally in compression class (CCL) 3.

##### Recommendation 9

In patients with UCV, treatment with MCS shall be tried as an alternative to PCB after decongestion.

##### Recommendation 10

When UCV patients are treated by compression with MCS, two-layer MCS systems designed for UCV should be used.

##### 2.1.2.2 Circumference.

Leg circumference is defined in sizes a to g, measured at length measurement points A to G. For mass-produced stockings, the standard requires a series of circumference measurements for slim, normal, and strong legs.

##### 2.1.2.3 Toe, heel, and hand.

MCS may have an open or closed toe. The toe area should be elastic, so as not to cause constriction. The heel, which is closed in all MCS, should also be elastic. Arm stockings can either include the hand or leave it exposed.

##### 2.1.2.4 Fit, ready-made and made-to-measure stockings, sizes.

MCS can only be effective if they fit properly, i.e., the length and circumference shall match the particular anatomy of the treated leg. Since the patient will wear the MCS when standing up, the size shall be measured in the standing position, especially in adipose patients. If both legs are to be fitted with MCS, each limb shall be measured individually.

If the measured length and circumference match a standard size, a ready-made stocking should be selected. Significant differences in the measurements often require the provision of a made-to-measure MCS. Flat-knit MCS should always be made to measure.

##### Recommendation 11

The limbs shall be measured when free of edema insofar as possible to ensure that the right size of MCS is selected. This applies to both ready-made and made-to-measure MCS.

##### 2.1.2.4.1 Reception of MCS.

The MCS shall be correctly received for correct issue to the patient.

##### Recommendation 12

On receipt, the following criteria at least shall be checked:

- Number
- Length: A–D calf stockings, A–F knee-length stockings, A–G thigh stockings, A–T compression support tights
- Compression class (CCL): I to IV
- Indication and diagnosis
- Device number or description
- Check made-to-measure production if necessary
- Toe: open or closed

Additional checks, if required:

- Made-to-measure production
- Auxiliary elements: e.g., fly opening, pelottes, zip closure, panty part with compression
- Flat knit
- Stiffness
- Closures: e.g., sticky tape, hook-tape (Velcro), waistband
- Resupply

As different materials are supplied for each compression class, with different elasticity and effectiveness, product information is often important. It is the physician’s job to find out the appropriate information.

##### 2.1.2.5 Pressure values and pressure profile.

MCS of compression classes (CCL) I, II, III, and IV differ in the intensity of the pressure applied to the limb at rest. The pressures required in the ankle area (B-measurements) are given in Table 1. Commercially available MCS apply a continuously reducing pressure from distal (ankle area) to proximal. Knee-length MCS with increasing pressure, and higher pressure in the calf than in the ankle area, have also been shown to be effective for CVI [5–7].

**Table 1:** Pressure in the ankle region according to RAL GZG 3872.2.6. Ordering of stocking type and compression class (CCL) [8]

CCL	Intensity	Pressure/mmHg	kPA
I	Slight	18–21	2.4–2.8
II	Medium	23–32	3.1–4.3
III	High	34–46	4.5–6.1
IV	Very high	49 and more	6.5 and more

The CCL are standardized based on the pressure at rest in the ankle area. This standardization is not international and the pressures in the individual classes may differ. However, the effectiveness of MCS is affected not only by the pressure at rest but also by the work pressure, and thus the material, which may be of different distensibility and elasticity (stiffness). A higher work pressure can be obtained either by a higher pressure at rest or by higher stiffness. There are therefore MCS of different materials in the different CCL. Putting on and taking off MCS by outpatient services is prescribable and refundable for all CCL on request.

##### Recommendation 13

The stocking type and the intensity of the pressure required, i.e., the CCL, are dependent on the diagnosis, the location of the drainage disturbance, the clinical findings, and the severity of the symptoms and alterations (e.g., severity of the edema). Strict attribution of a CCL to a diagnosis is meaningless. The object of compression therapy is to improve the clinical findings.

##### Recommendation 14

The lowest effective CCL shall always be preferred. This supports adherence to compression therapy.

CCL I may be sufficient to clear up the symptoms of a varicose vein or a pronounced edema, while a higher CCL will be needed with a more advanced edema and skin alterations. CCL II is usually sufficient for the early stages of post-thrombotic syndrome, while higher CCL and short-stretch materials may be needed for more severe stages. When a lymphedema is starting (stage I), CCL II is usually sufficient, while in stage III, the higher pressure of CCL III or even IV may be necessary.

If the patient is not in a physical condition to put on MCS of higher CCL (III and IV) by him-/herself, the alternative of wearing two stockings of lower CCL, one over the other, is recommended. Another alternative is to put on individual elements one over another, e.g., forefoot cap, knee-length stocking (A–D), and capri (C–T), or thigh stocking (A–G) and shorts (F–T; with fly opening and/or zip as required). Zips can also be incorporated to order to make the stockings easier to put on and take off. The skin can be protected with an understocking.

##### 2.1.2.6 Materials.

Unstretched MCS are 0.5 to 1.4 mm thick and made of variable combinations of polyamide, elastane, cotton, elastodiene, rayon, or microthreads.

- *Polyamide (PA, Nylon®, Perlon®)*
  Polyamide thread fabrics are resistant to ageing, insects, rot, moths, and microorganisms. The maximum moisture content is 0.06%.
- *Elastane (EL, Lycra®)*
  This material consists of 85% polyurethane (PU). The highly elastic threads are resistant to almost all diluted acids and alkaline, as well as oil and grease. They resist ageing, light and temperatures up to 150 °C. The moisture absorbance is very low, 1.5%.
- *Cotton (CT)*
  Cotton is a fiber which covers the seeds of the cotton plant (Malvaceae), an annual shrub which usually grows 2 to 3 m high. Raw CT normally contains 83–85% pure cellulose. CT can be boiled and sterilized; static charging is low. Its elasticity is approximately 40%, and the moisture content under normal conditions is 8%.
- *Elastodiene (ELA, natural rubber)*
  The raw material is the sap of the rubber tree (natural latex), harvested by cutting slits in the bark. ELA is remarkable for its extraordinarily high elasticity and distensibility. It is not resistant to grease or many chemical substances. It is destroyed by high temperatures (e.g., for sterilization). The moisture absorbance is low.
- *Rayon (CV, viscose)*
  In contrast to cotton, rayon is a regenerated cellulose thread, a chemical thread. Its moisture content varies between 5 and 15% and it is used as a substitute for cotton. Like cotton, it has high moisture absorbance (swelling capacity 85–120%).
- *Microthreads*
  This is a general description for polyamide and polyester threads (moisture content 0.5–4%). They are substantially thinner than natural threads, with a fineness of 0.1–1 dtex, i.e., 10,000–100,000 m of thread weigh 1 g. Well-known materials made of microthreads are Tactel® and Trevira Finesse®.
- *Colors, patterns, and trimmings*
  Both flat- and circular-knit MCS are available in a wide range of colors, which helps to make therapy more acceptable. There are also MCS in different patterns, with different types of knit and trimmings.
- *New developments—auxiliary elements*
  To increase wearer comfort and adherence, many MCS include ceramic capsules woven into the stocking which release conditioning substances. In other models, the actual threads are enriched with conditioning substances. If necessary, they can be re-impregnated after the stocking has been washed a few times. MCS are also available with silver-coated threads to reduce bacterial colonization of the skin.

##### 2.1.2.7 Tolerance.

MCS and PCB are generally well tolerated. Allergies to polyamide, elastane, cotton, rayon, or microthreads in the form of urticaria (immediate allergy) or contact eczema (delayed allergy) are very rare. Allergies to latex or rubber ingredients occur more frequently, but these are no longer used in modern MCS, except in the borders. Allergies may occur due to the disperse dyes DP blue 124 and DP blue 106, used to obtain dark colors like dark blue and black; however, these dyes are prohibited in the EU [9].

Wearing an MCS or a PCB should not alter the physiological pH values of the skin.

According to the fifth amendment to the Consumer Goods Ordinance (German law), MCS can no longer contain disperse (azo) dyes which release carcinogenic amines (arylamine). The use of chrome-IV compounds and certain flameproof products is banned. Because of their high allergenicity, the Federal Institute for Health Protection of Consumers and Veterinary Medicine advises against the use of disperse dyes: disperse blue 1, 35, 106, 124; disperse yellow 3; disperse orange 3, 37/76, and disperse red. MCS containing cotton may not contain prohibited pesticides. The established maximum permitted values for insecticides, herbicides, pyrethroids, and chlorophenols shall not be exceeded.

##### 2.1.2.8 Durability.

MCS are usually designed to carry out their medical effect (compression) for a useful life of 6 months. This depends on correct treatment of the stocking (e.g., product care, donning, and doffing) and normal use.

##### Recommendation 15

MCS should be used to carry out their medical effect (compression) for a useful life of 6 months. This depends on correct manipulation of the stocking (e.g., product care, donning, and doffing) and normal use. However, wear and tear due to professional or medical requirements may lead to significant alterations in durability. If the MCS wears out sooner due to professional or medical requirements, or a pronounced change in the shape of the leg, a new one can be ordered earlier. When the first MCS is ordered, a second should always be ordered at the same time for hygienic reasons.

##### 2.1.2.9 Labelling.

Unambiguous, lasting labelling of the MCS guarantees correct classification even after a long period of use.

##### Recommendation 16

Every MCS should have a durable label with the following information:

- Producer
- Product name
- Fiber content
- Compression class
- Stiffness
- Stocking type or size
- Product care and washing symbols

MCS are classified in medical product laws as risk class I, i.e., the producer can attach the CE symbol to the MCS once the guidelines are met. MCS are included in the Universal Medical Device Nomenclature System (UMDNS) under number 13-789 [10]. Production in compliance with CE criteria alone does not guarantee the requirements necessary for medical treatment at the correct dosage.

##### 2.1.2.10 Care.

Correct care of the MCS is indispensable for maintaining its effectiveness.

##### Recommendation 17

The MCS should be washed daily, as sweat and dirt deteriorate the material. The producer will provide indications on the washing and care of the garment.

#### 2.1.3 MCS donning and doffing aids

Appliances to assist in donning and doffing MCS help to protect and preserve the stocking material, and make these actions easier. They allow patients with restricted movement to handle the materials by minimizing the movement and strength that they shall exert. Donning and doffing appliances, like MCS and ulcer stocking systems, have an appliance authorization. There are models for open and closed MCS. They include slides, frames, and special forms. Donning and doffing aids shall be ordered on a separate prescription with the appropriate indications and diagnosis.

*Indications for ordering donning and doffing aids include:*

- Paralysis
- Age-induced loss of strength
- Arthrosis/rheumatism
- Severe obesity
- Extensive stiffness of the spine/hip/knee
- Degenerative diseases in the hands or hand region
- Sequelae from injuries/amputations

##### Recommendation 18

In cases of restricted movement and problems in donning and doffing the MCS, the appropriate aids should be ordered.

### 2.2 Phlebological compression bandage (PCB)

#### 2.2.1 Bandage types

Reusable elastic materials shall be distinguished from non-reusable materials, such as adhesive (sticky) bandages and the barely distensible zinc paste bandages.

Short-stretch, medium-stretch, and long-stretch bandages shall also be distinguished. In addition to zinc paste bandages, there are padded and self-adhesive bandages. Multiple-component systems are prepared sets containing several of these materials in combination. Compression bandages are applied according to the patient’s capabilities and acceptance, as well as medical indications.

##### Recommendation 19

The selection of the compression bandages used to make up a PCB should follow the operator’s knowledge, the patient’s preferences, the medical indications, and the type of bandage.

#### 2.2.2 Materials

Compression bandages are principally made of polyamide, elastane, cotton, elastodiene, and rayon in different combinations.

*Polyamide (PA, Nylon, Perlon):* Polyamide thread fabrics are resistant to ageing and microorganisms. The maximum moisture content is 0.06%.

*Elastane (EL, Lycra®):* This material consists of 85% polyurethane (PU). The highly elastic threads are resistant to oil and grease. They resist ageing, light, and temperatures up to 150 °C. The moisture absorbance is very low, 1.5%.

*Cotton (CT):* CT is a natural product of plant origin. Raw CT contains 83–85% pure cellulose. CT can be boiled and sterilized; static charging is low. Its distensibility is approximately 40% and the moisture content under normal conditions is 8%.

*Elastodiene (ELA, natural rubber):* The raw material is the sap of the rubber tree (natural latex). ELA is remarkable for its extraordinarily high elasticity and distensibility. It is not grease resistant. It is destroyed by high temperatures (e.g., for sterilization). The moisture absorbance is low.

*Rayon (CV, viscose):* In contrast to cotton, rayon is a regenerated cellulose thread, a chemical thread. Its moisture content varies between 5 and 15% and it is used as a substitute for cotton. Like cotton, it has high moisture absorbance (swelling capacity 85–120%).

#### 2.2.3 Bandage width and length

Compression bandages are available in widths of 6, 8, 10, and 12 cm, and lengths of 5, 6, and 7 m.

#### 2.2.4 P-LA-C-E

The acronym P‑LA-C‑E represents a widely used concept for the evaluation of compression bandages [11]. They are evaluated for:

- P (pressure)—exerted by the compression bandage on the limb;
- LA (layers)—wrapping of the material over itself, whether it consists of one or more components;
- C (components)—type of materials used in the manufacture of the individual components; and
- E (elasticity)—enabling the material to apply high pressure to the limb at rest.

#### 2.2.5 Material tolerance

PCB are well tolerated as a rule. Allergies to polyamide, elastane, cotton, elastodiene, and rayon in the form of urticaria (immediate allergy) or contact eczema (delayed allergy) are very rare. Latex or rubber ingredients are seldom used in compression bandages. Wearing a PCB should not alter the physiological pH values of the skin.

The availability of colored bandages has increased in recent years. Because of their high allergenicity, the Federal Institute for Health Protection of Consumers and Veterinary Medicine advises against the use of disperse blue 1, 35, and 106, disperse yellow 3, disperse orange 3, 3/76, and disperse red dyes. According to the Consumer Goods Ordinance, products may not contain azo dyes which release carcinogenic amines (arylamine). The use of chrome-IV compounds and certain flameproof products is banned.

#### 2.2.6 Labelling

Unambiguous, lasting labelling of the bandage guarantees correct classification even after a long period of use.

##### Recommendation 20

Reusable bandages should have a durable label with the following information: producer, product name, main components, length (unstretched), width (unstretched), distensibility, product care, and washing symbols. Single-use bandages should carry the information marked on the packaging, e.g., recognizable by a figure 2 in a circle crossed-out.

#### 2.2.7 Distensibility

Bandages are mostly made to stretch longitudinally. A consensus paper [11] recommends that bandages should be distinguished between inelastic, with distensibility of less than 100%, and elastic, with distensibility of more than 100%. Distensibility describes the property of the material to become longer when pulled. Long-stretch bandages are highly distensible while short-stretch bandages are only slightly distensible. The point to which the bandage can stretch is called the distensibility point. In long-stretch bandages the distensibility is between 140 and 200%. Bandages with lower distensibility, between 10 and 100%, are called short-stretch bandages. Zinc paste bandages, with distensibility of less than 10%, are a special form.

Some bandages are elastic both lengthwise and crosswise. Knowledge of the crosswise elasticity is of interest insofar as different lengthwise distensibility may be combined with different crosswise distensibility. Experience shows that a PCB made with dual-tension bandage “sits” better than one made with lengthwise elastic bandage.

#### 2.2.8 Durability

The minimum durability shall be stipulated in reusable bandages.

##### Recommendation 21

Reusable compression bandages should be able to be washed (boiled, steam-sterilized, chemically sterilized) at least 15 times before their elasticity starts to diminish perceptibly.

#### 2.2.9 Pressure values and pressure profile of PCB

The application of constant pressure by the bandage depends on several factors:

- Distensibility
- Type
- Width
- Elasticity
- Relaxation
- Number of wraps
- Bandaging technique
- Moisture absorbance
- Care and washing
- Circumference of limb
- Configuration of limb

When a PCB is applied, the pressure should diminish from distal to proximal. The following practical points should be considered: pressure, number of layers, material components, and elastic properties [11].

#### 2.2.10 Desired local pressure

There is good evidence that higher pressure is better, e.g., for curing a venous leg ulcer, than lower pressure. The PCB should be applied to UCV patients with a high pressure, i.e., ≥40–60 mmHg. In accordance with an international consensus of the International Compression Club (ICC), the following classes of compression pressure values are recommended for compression bandaging [11]:

- Slight: <20 mmHg
- Medium: ≥20–40 mmHg
- High: ≥40–60 mmHg
- Very high: >60 mmHg

This classification is similar to the compression classes for MCS; however, the value ranges are different. Both classifications use the same concepts to describe the intensity: slight, medium, high, and very high. When bandage systems are applied, the real pressure applied can only be estimated and depends on the experience and practical abilities of the operator. The pressure under the compression bandage can be measured with an electronic pressure-measuring device applied to the B1 region.

#### 2.2.11 Bandaging techniques

There are many bandaging techniques, most of which have names which refer precisely to the manner of proceeding. A distinction is also made between systems consisting of one or several component materials; in the latter case, each individual component is usually applied by wrapping several layers over each other.

##### Recommendation 22

The following important principles shall be observed when applying a PCB to the leg:

- Each layer of the bandage system shall be wrapped over the preceding layers.
- The ankle should be positioned at a right angle (dorsiflexion).
- In the lower leg, the compression bandage is applied up to the head of the fibula; in the thigh, up to the proximal thigh.
- Due to the geometry of the leg, the pressure applied with the same distension of the bandage reduces as the radius increases from distal to proximal.
- The bandage shall not be applied with points of high pressure, strangulating, or constrictions, or cause pain.
- The PCB material and application technique shall be adapted to the requirements of the patient’s medical condition.

##### 2.2.11.1 Basics of bandaging.

It has not been shown that any particular bandaging technique, e.g., those proposed by Pütter, Sigg, or Fischer, is superior to the others. The correct, competent execution of the method chosen is decisive for efficient bandaging [12].

##### Recommendation 23

Care should be taken with the following aspects of compression bandaging:

- A cotton tubular bandage down to below the knee serves to protect the skin.
- Inner padding can help to prevent pressure ulcers.
- Pressure padding and pelottes can increase the effectiveness of bandaging.
- Fixing clips often present a risk of injury; they are only to be used to fix the bandage outside the padding, not on the inside (see supplier’s information). Strips of sticking plaster are suitable for fixing the tail of the bandage.
- The width of the bandage is adapted to the shape and diameter of the limb.
- At least two bandages are normally required to obtain the required compression.
- The foot should always be in its physiological position (dorsiflexion).
- Ensure that proper pressure in each layer is applied from the start. Slack turns, for example on the front part of the foot, can lead to the formation of edemas.
- The roll of bandage is kept under permanent tension and unrolled directly onto the skin, so that the bandage follows the contours of the leg smoothly.
- Pulling individual turns too tight disturbs the pressure gradient. Strangulating constriction can lead to vein congestion (and even an increased risk of thrombosis), pressure damage to nerves, or necrosis.
- In cases of pronounced edema of the front part of the foot, or pronounced lymphedema, the toes shall also be compressed to avoid influence of the edema.

##### 2.2.11.2 Padding.

Incorrect application of a PCB risks causing pressure-induced damage, which can lead to pressure ulcers, skin necrosis, and nerve damage [13]. Even bandaging applied by a qualified expert can lead to unnoticed constriction, nerve damage, or blisters. Appropriate inner padding beneath the bandage with cottonwool or foam bandages and/or pelottes prevents these unwanted side effects. The shape of the bandaged limb shall also be considered during application, ensuring that any bulges or hollows are padded. In this way the desired pressure can be applied uniformly to the whole leg.

The same is true of the upper limbs.

The impact of the bandaging technique on the pressure applied is disputed. Coull and colleagues compared PCB in which the bandage was applied with figure-of-eight turns vs. spiral turns, and measured that a higher pressure could be achieved with the former [14]; however, spiral turns produced a higher pressure in a work by Lee et al. [15].

#### 2.2.12 Care

Care of reusable bandages is important, since it affects their quality and therefore their therapeutic effectiveness. Bandages are highly sensitive to grease, oil, and creams. Bandages containing rubber in particular are damaged by these materials, which reduce their elasticity. The patient is recommended to wash the bandages daily on hygienic grounds, since sweat and dirt attack the material and remains of exudates can foster infections. The producer will provide indications on the washing and care of the garment.

#### 2.2.13 Materials for the decongestion phase

Many patients with venous or lymphatic pictures have an uncompensated edema at the start of treatment. In these cases, an initial decongestion phase is indicated.

PCB consisting of reusable compression bandages, especially short-stretch bandages, are quickly loosened by movement; they slip and lose their shape within a few hours, so they do not maintain the desired pressure for long. Furthermore, the pressure diminishes substantially in the first 24 h due to movement and the reduction of edemas [16, 17]. As a result, bandaging with these materials shall be reapplied daily if necessary in the initial decongestion phase. There may also be serious failings by the operator in correct application of the bandage [18].

Multiple-component systems are designed for use by people with venous leg ulcers (UCV). They consist of two to four components, e.g., padding, compression, and cohesive fixing bandages. These systems have the advantage over short-stretch bandages that no elaborate compression techniques have to be mastered. Some products have optical markers or predefined distensibility. This makes it easier to obtain the right therapeutic pressure. Correct application should produce a pressure of 40 mmHg, the value indicated for treating an active UCV. Patients with arterial circulation disturbances but without critical ischemia, with an ABI between 0.6 and 0.8, can be treated with so-called lite versions of this system. Multiple-component systems are left on for up to 7 days. The final cohesive bandage prevents slipping, and the application pressure is maintained continuously until the next bandage change, subject to the decongestion situation. Multiple-component systems produce decongestion significantly more quickly, are easier to apply, and offer the patient greater comfort than PCB with short-stretch bandages. Furthermore, the patient has greater ankle movement, which improves the venous pump [12]. After the initial decongestion phase, in certain cases the treatment should be changed from bandages to a two-layered ulcer compression stocking system for long-term therapy [19].

##### Recommendation 24

In the decongestion phase, multi-layered compression bandages can be used, or else multiple-component systems, which have proved effective especially in UCV patients.

##### Recommendation 25

The selection of the bandaging technique or bandage system should be based on the diagnosis, the symptoms, and the patient’s preferences, as well as on the experience and competence of the operator.

##### Recommendation 26

After the initial decongestion phase, in certain UCV patients the treatment should be changed from PCB to a two-layered ulcer compression stocking system for long-term therapy.

#### 2.2.14 Adherence

Some studies describe better adherence with MCS than PCB [20–22].

### 2.3 Medical adaptive compression systems (MAC)

New compression systems have been available for a few years which should minimize the application problems experienced by individual patients with the compression systems available previously [23]. These compression systems are used in the decongestion phase. Like short-stretch bandages, MAC provide a high working pressure and a low rest pressure. In contrast to bandaging systems, loss of pressure can be corrected by adjusting the clips during use, which helps in the remission of edemas. Because their application is significantly simpler, these systems require less time to apply and the probability of making a mistake is lower than with more elaborate compression bandaging systems [12]. Patients who are still sufficiently mobile, or whose household members are able, can often apply the MAC themselves after a short introduction. This also improves adherence. Such systems, with their clips, can be donned and doffed—and adjusted as the edema reduces—relatively easily and independently by sufficiently mobile patients. MAC are available in different sizes, so they can be matched to the patient’s measurements in advance as well as being adjusted at the time of application. There are different systems for treating different indications, like lymphedemas, phlebological edemas, or venous ulcers [24–26]. Commercially available materials are characterized by relatively high stiffness, which can contribute substantially to their effectiveness. Like MCS, MAC are available in three different sizes: short, normal, long.

MAC can be used alone or in combination with an MCS. Patients can manage these systems themselves, as they have reproducible pressures [27] and generally a high level of stiffness. A comprehensible classification system, such as exists for two-layered ulcer-compression stocking systems or MCS, is lacking and needs to be worked out.

#### Recommendation 27

In the initial decongestion phase with lymphedema and pronounced venous edema, as well as in UCV treatment, MAC can be applied as an alternative to bandaging.

## 3 Indications for medical compression therapy

### Recommendation 28

The following indications for medical compression therapy shall be heeded:

*Chronic vein diseases:*

- Improvement of venous symptoms
- Improvement in quality of life in chronic vein diseases
- Prevention and treatment of venous edema
- Prevention and treatment of venous skin alterations
- Eczema and pigmentation
- Dermato-liposclerosis and *atrophie blanche*
- Venous leg ulcer treatment
- Mixed (arterial and venous) leg ulcer (respecting contraindications: see Recommendation 31)
- Prevention of recurrent venous leg ulcer
- Pain reduction in venous leg ulcer treatment
- Varicose veins
- Initial phase after varicose vein therapy
- Functional venous incompetence (cases of obesity, people who work sitting/standing for extended periods)
- Venous malformations

*Thromboembolic venous diseases:*

- Superficial venous thrombosis
- Deep leg venous thrombosis
- Arm vein thrombosis
- Condition after thrombosis
- Post-thrombotic syndrome
- Thromboprophylaxis in mobile patients

*Edema:*

- Lymphedema
- Edema during pregnancy
- Post-traumatic edema
- Post-surgery edema
- Post-surgery reperfusion edema
- Idiopathic cyclic edema
- Lipedema from stage II
- Congestion following immobility (arthrogenous congestion syndrome, paresis, and partial paresis of the limb)
- Work-induced edema (people who work sitting/standing for extended periods)
- Medication-induced edema, when substitution is impossible

*Other indications:*

- Obesity with functional venous incompetence
- Inflammatory skin diseases on the legs
- Nausea and dizziness in pregnancy
- Symptoms of congestion during pregnancy
- Condition after a burn
- Scar treatment

The effectiveness of compression therapy in the individual indications is discussed in Sect. 8 “Effectiveness of medical compression therapy.” The indication for compression therapy in the initial and follow-up treatment of deep leg vein thrombosis was undisputed in earlier studies [28, 29], but has been questioned in more recent studies [30, 31]. The OCTAVIA and IDEAL studies have recently confirmed that compression therapy with good adherence can significantly reduce the incidence of PTS [32, 33]. The latest outcomes from the IDEAL study have also shown a reduction in the incidence of PTS when compression therapy is started immediately compared to delayed start [34]. In view of the above, the following is recommended:

### Recommendation 29

If deep leg vein thrombosis is diagnosed, compression therapy shall be started immediately.

### Recommendation 30

After deep leg vein thrombosis, compression therapy should be continued for at least 6 months. Thereafter the continuation and duration of compression therapy should be decided according to the subjective and objective signs of post-thrombotic syndrome (e.g., pain, feeling of heaviness, edema, skin alterations).

## 4 Contraindications for phlebological compression therapy [35]

### Recommendation 31

The following contraindications for medical compression therapy shall be heeded:

- Advanced peripheral arterial occlusive disease (if any of these parameters is exceeded: ABPI <0.5, arterial pressure at the ankle <60 mmHg, toe pressure <30 mmHg, or TcPO2 <20 mmHg at the back of the foot). If inelastic materials are used, compression treatment can still be attempted with arterial pressure at the ankle between 50 and 60 mmHg under close clinical supervision [36].
- Decompensated heart failure (NYHA III + IV)
- Septic phlebitis
- Phlegmasia cerulea dolens

### Recommendation 32

The following risks for medical compression therapy shall be heeded:

- Pronounced weeping skin changes
- Intolerance to compression material
- Severe sensitivity disturbances of the limb
- Advanced peripheral neuropathy (e.g., with diabetes mellitus)
- Primary chronic polyarthritis

In these cases, the decision on treatment should be taken based on a balance of the benefits and risks, and selection of the most suitable compression device.

## 5 Risks and side effects

Incorrect bandaging (excessive pressure, strangulation) causes pain and can lead to tissue damage and even necrosis and pressure damage to peripheral nerves, especially at bone protrusions (cave, e.g., fibula heads) [37, 38]. MCS can cause skin necrosis and pressure damage to nerves, especially with incorrect application. Sensitive skin may suffer itching, scaling, and signs of inflammation under compression devices. Proper skin care is therefore recommended with compression treatment.

### Recommendation 33

To avoid the side effects and risks of compression therapy, the rules for correct application shall be observed. This includes padding of areas at risk from pressure and regular skin care.

### Recommendation 34

If any of the following symptoms are observed, the compression device shall be removed immediately and the clinical findings examined: blue or white discoloration of the toes, paresthesia and numbness, increasing pain, shortness of breath and sweating, acute limitations on movement.

## 6 Physical activity for diseased veins

Compression therapy is most effective with regular activation of the muscle pump. The patient’s movements are therefore an integral part of effective compression therapy. Patients are encouraged to carry out regular foot exercises and to walk [39, 40].

## 7 Education

The patient’s adherence to the therapy and acceptance of necessary treatment options and operations are decisive for successful treatment [41]. This includes correct use and care of the necessary compression materials, and the patient’s ability and willingness to care for his/her skin and carry out physical activity for diseased veins. Individual explanation and education allow the patient to develop understanding of the disease and the measures applied to combat it [42]. The content should always be adapted to the patient’s individual needs [35]. Leaflets are a useful complement to patient education; they are an effective way of increasing patient satisfaction and adherence and can contribute to the strengthening of many patients [43]. A well-informed patient can contribute constructively to the treatment as an equal partner.

### Recommendation 35

To encourage adherence, the patient shall be thoroughly educated in the purpose and logic of compression therapy, possible side effects and risks, and the correct use of compression materials and associated measures (e.g., skin care, exercise).

## 8 Effectiveness of medical compression therapy

MCS keep the compression pressure at the correct level for many hours [16, 44]. Even after a full working day, the MCS does not lose its elastic force. In individual studies, adherence was better with MCS than with compression bandages [20].

Clinical studies contain numerous proofs of the effectiveness of compression therapy [45].

### 8.1 Chronic venous diseases

#### 8.1.1 Improvement of symptoms, quality of life, and edemas

Various studies have shown an improvement in vein symptoms, quality of life, and venous edemas, even with low pressures, as compared to placebo stockings [46, 47]. A significant reduction in edemas and symptoms has also been shown in people who work standing up [48, 49], after long-haul flights [50], in aircrews [51, 52], in chronically immobilized elderly people after sitting still for extended periods [53], and in patients with intradermal varices [54] and CVI [6, 7]. MCS improve the symptoms of varicose veins in pregnancy [55, 56]. Symptoms and edemas do not always need to be treated with high pressures; low pressure can also be effective [57, 58]. Where this is possible, treating varicose veins by sclerotherapy or an operation is more effective than compression therapy [54, 59]. Even the painful symptoms of venous leg ulcer patients can be significantly improved with compression therapy [60]. Compression bandages and two-layered ulcer compression stocking systems improve ulcer healing and reduce pain in equal measure [61].

#### 8.1.2 Improvement of skin alterations

Compression therapy is applied in the daily routine to improve skin alterations in patients with venous and lymphatic diseases. Vandongen showed improvements in lipodermatosclerosis with severe CVI [62].

#### 8.1.3 Prevention of recurrent venous leg ulcer

Nelson investigated the effect of compression therapy on recurrent venous leg ulcer [63]. Wearing MCS of different CCL reduced the recurrence rate significantly. Moderate compression resulted in better adherence than high compression. In 2006, Nelson compared the effectiveness of MCS with low (18–24 mmHg) and high (25–35 mmHg) compression on the recurrence rate of recently healed venous ulcers with a follow-up of 5 years [64]. He found no significant differences. In 2007, Gohel showed that compression therapy combined with operative treatment of varices resulted in no improvement in the healing time of venous ulcers as compared to compression therapy alone, but that the recurrence rate could be significantly reduced by the operation [65]. In 2014, Clarke-Moloney randomized 100 patients with healed UCV into two groups, one with MCS of CCL I (18–21 mmHg) and the other with MCS of CCL II (23–32 mmHg) [66]. After 12 months’ follow-up, no significant difference was found in the recurrence rate. Patients who abandoned compression therapy presented a significantly higher recurrence rate. Kapp compared MCS of CCL II (23–32 mmHg) and CCL III (34–46 mmHg) for prophylaxis of ulcer recurrence in patients in a nursing home [67]. The risk of recurrence was higher with CCL II, but adherence to the compression therapy was low at 44%. With CCL III the adherence was significantly worse, and the patients with no compression therapy presented the highest recurrence rate.

#### 8.1.4 Improvement of ulcer healing

PCB are recognized as the standard treatment for venous leg ulcers, but MCS and MAC are also both suitable for ulcer therapy [68]. Blecken and colleagues showed that treatment with MAC produced a reduction in wound size significantly faster than a four-component PCB [26]. As early as 1994, Partsch and Horakova showed that MCS and PCB produced comparable healing of leg ulcers [69]. Jünger, in 2004, showed that special two-layered ulcer compression stockings led to better healing than standard PCB [70]. Ashby randomized 457 ulcer patients into one group with a four-component PCB and one with a two-layered ulcer compression stocking system [71]. The healing rate was practically identical (70.9% with the ulcer compression stocking system and 70.4% with four-component PCB).

#### 8.1.5 Reduction of side effects after invasive varicose vein treatment and improvement of outcomes

Compression therapy is established in many guidelines as part of the posttreatment after invasive varicose vein treatment. The majority of current studies show a reduction of pain and edemas in the first week after crossectomy and stripping or endovenous thermal ablation of the great saphenous vein [72–76]. Eccentric pressure increase along the treated vein improves the outcome [77–79]. Hamel-Desnos found no significant difference for pain and complications after foam stripping of the great saphenous vein between MCS (15–20 mmHg) and no-compression therapy [80].

An improvement in the clinical outcome achieved by compression therapy has only been described for spider vein ablation. Kern reported a better outcome after spider vein ablation when MCS (23–32 mmHg) were worn for 3 weeks [81]. Weiss reported less pigmentation after sclerosis therapy when compression therapy was applied [52].

Not all patients reach a symptom-free state after invasive treatment; many continue to have signs or symptoms of CVI. In these cases, continued long-term compression therapy is indicated.

### 8.2 Acute venous diseases

#### 8.2.1 Deep leg vein thrombosis

In cases of acute deep leg vein thrombosis (DVT), compression therapy has a long history as a complement to anticoagulation [82]. It plays a particularly important role in early mobilization and ambulatory treatment of DVT [83, 84].

##### 8.2.1.1 Initial reduction of pain and edema.

Various studies have shown that immediate compression therapy and mobilization after diagnosis lead to a faster reduction of pain and edemas in the affected leg than no compression therapy [85, 86]. The ability to walk a longer distance without pain is also linked to this treatment [85]. Compression therapy started after 2 weeks or more has no additional effect on initial pain reduction [30, 31].

##### 8.2.1.2 Mass reduction of thrombus.

Arpaia et al. show that patients treated with compression therapy immediately after diagnosis presented significantly faster recanalization of the thrombus than patients who only started compression therapy 2 weeks after diagnosis [87]. Blättler proved less progression of the thrombus in the compression group [85].

#### 8.2.2 Superficial vein thrombosis

In a current Cochrane Review on the treatment of superficial vein thrombosis (SVT), compression therapy was adopted as a standard treatment in addition to other measures [88]. Böhler found no additional effect of compression therapy with thigh-length stockings (21–32 mmHg) on painful symptoms in SVT patients who routinely received anticoagulation with low-molecular-weight heparin and non-steroidal anti-inflammatory drugs [89]; however, significantly faster thrombus regression in the first weeks under compression therapy was reported.

#### 8.2.3 Prophylaxis of immobilization-induced thromboembolic complications

A calf stocking with low to medium compression reduces the risk of deep leg vein thrombosis on long-haul flights significantly [90, 91]. Contradictory outcomes are reported on the occurrence of superficial vein thrombosis on long-haul flights [90, 91]. For thromboembolic prophylaxis in the hospital, in the context of an operation or immobilization due to disease, we refer to the S3 guidelines for the prophylaxis of venous thromboembolism (VTE; AWMF 003-001, S3).

### 8.3 Post-thrombotic syndrome (PTS)

#### 8.3.1 Prophylaxis of PTS

Post-thrombotic syndrome (PTS), with pains, edema, and skin alterations, and even UCV, is a frequent sequela of DVT [92]. The Villalta score is used to quantify PTS [93, 94]. Earlier studies showed halving of the incidence of PTS under compression therapy for 2 years [28, 29]. Thigh-length MCS were no more effective than knee-length stockings for prophylaxis against PTS [95]. Compression therapy over a long period reduced the symptoms associated with PTS [96]. These positive outcomes were not found in the SOX study, in which compression therapy was only started 2 weeks after diagnosis, and the adherence to compression therapy was low [30, 31]. The effectiveness of compression therapy with good adherence as prophylaxis against PTS was shown again in the recently published OCTAVIA and IDEAL studies [32, 33]. A sub-study of the IDEAL study also showed that starting compression therapy immediately rather than after 2 weeks led to significantly lower objective PTS parameters in the Villalta score [34].

#### 8.3.2 Treatment of PTS

Symptoms and skin alterations caused by PTS, including UCV, can be made better with compression therapy [97–99]. Lattimer also showed improved hemodynamics with compression therapy in PTS patients [100].

### 8.4 Lymphedema

Insufficient data are available on prophylaxis against lymphedema after a tumor operation. Compression therapy is a constant component of the complex physical decongestion therapy of lymphedemas. It is useful both for reducing the edema and for keeping the patient edema free. It can be applied with or without manual lymph drainage [101–104].
